# RNA Modification in Inflammatory Bowel Diseases

**DOI:** 10.3390/biomedicines10071695

**Published:** 2022-07-13

**Authors:** Mika Nakayama, Yuki Ozato, Yoshiko Tsuji, Yasuko Arao, Chihiro Otsuka, Yumiko Hamano, Genzo Sumi, Ken Ofusa, Shizuka Uchida, Andrea Vecchione, Hideshi Ishii

**Affiliations:** 1 Department of Medical Data Science, Center of Medical Innovation and Translational Research, Graduate School of Medicine, Osaka University, Suita 565-0871, Japan; nakayama@cfs.med.osaka-u.ac.jp (M.N.); catcherweek927@gmail.com (Y.O.); tsuji@cfs.med.osaka-u.ac.jp (Y.T.); arao@cfs.med.osaka-u.ac.jp (Y.A.); cot@cfs.med.osaka-u.ac.jp (C.O.); hamano@cfs.med.osaka-u.ac.jp (Y.H.); hisho@kinshukai.or.jp (G.S.); oof21443@ideacon.co.jp (K.O.); 2 Department of Gastrointestinal Surgery, Graduate School of Medicine, Osaka University, Suita 565-0871, Japan; 3 Kinshu-kai Medical Corporation, Osaka 558-0011, Japan; 4 Food and Life-Science Laboratory, Prophoenix Division, IDEA Consultants, Inc., Osaka 559-8519, Japan; 5 Center for RNA Medicine, Department of Clinical Medicine, Aalborg University, Frederikskaj 10B, 2. (Building C), 2450 Copenhagen SV, Denmark; suc@dcm.aau.dk; 6 Department of Clinical and Molecular Medicine, University of Rome “Sapienza,” Santo Andrea Hospital, Via di Grottarossa, 1035-00189 Rome, Italy; andrea.vecchione@uniroma1.it

**Keywords:** RNA modification, inflammatory bowel disease

## Abstract

Inflammatory bowel disease (IBD) is a chronic inflammatory disorder characterized by damage to the intestinal mucosa, which is caused by a combination of factors. These include genetic and epigenetic alterations, environmental influence, microorganism interactions, and immune conditions. Some populations with IBD show a cancer-prone phenotype. Recent studies have provided insight into the involvement of RNA modifications in the specific pathogenesis of IBD through regulation of RNA biology in epithelial and immune cells. Studies of several RNA modification-targeting reagents have shown preferable outcomes in patients with colitis. Here, we note a new awareness of RNA modification in the targeting of IBD and related diseases, which will contribute to early diagnosis, disease monitoring, and possible control by innovative therapeutic approaches.

## 1. Introduction

**Figure 1:**
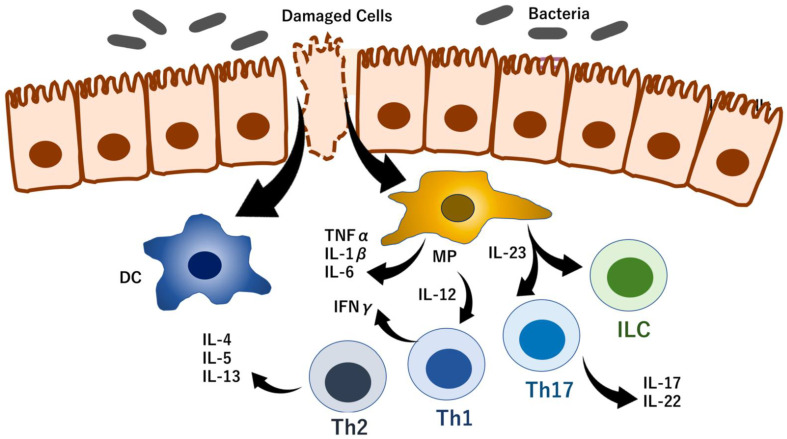
Overview of the mechanism of IBD. IBD is a complex disease that develops as result of multiple factors. Previous studies have revealed that genetic and epigenetic factors are involved in the maintenance of epithelial cell homeostasis in response to intestinal bacteria, food intake, and several irritants (e.g., cigarette smoking and alcohol intake), leading to epithelial cell damage and inflammation. Cell damage and infections stimulate dendritic cells (DC) and macrophages (MP) to secrete cytokines, including IL23, resulting in the activation of the immune response of innate lymphoid cells (ILC). The immune response can induce epithelial cell damage. In IBD, inhibition or modulation of the hyper reaction of immune cells or cytokines is a therapeutic target. In this review article, we focus RNA modification in IBD, as mentioned text, figures and tables.

Although recent studies have described substantial advances in our understanding of the molecular pathogenesis of inflammatory bowel disease (IBD), the mechanism remains unclear. In terms of etiology, IBD comprises a group of chronic inflammatory diseases [e.g., ulcerative colitis (UC) and Crohn’s disease (CD)] characterized by damage to the intestinal mucosa, which is believed to be caused by a combination of factors, including abnormal immunity of the intestinal mucosa [1]. In accordance with the abnormal autoimmune function in patients with IBD, the gut and other organs and tissues may be damaged [2] and may be susceptible to cancer development [3]. Recent studies have shown that various factors are involved in and are functionally integrated into the pathogenesis of IBD. First, the occurrence and development of IBD involve genetic factors, including germline genetic background, such as the presence of IBD-causative and/or IBD-associated variants, including rare variants in nucleotide-binding oligomerization domain containing 2 (*NOD2*), interleukin-23 receptor (*IL23R*), caspase recruitment domain family member 9 (*CARD9*), E3 ubiquitin-protein ligase, ring finger protein 186 [RNF186], and adenylate cyclase 7 (*ADCY7*) [4]. Second, environmental influences (e.g., dietary fiber, fruits, vegetables, and saturated fats, depression and impaired sleep, and low vitamin D levels) have all been associated with incident IBD [5]. Third, intestinal epithelial glycosylation is altered in IBD, in which, truncated O-glycans and terminal glycan structures are abnormally expressed due to mislocalization and altered expression, leading to the disruption of the mucus layer, glycan-lectin interactions, and host-microorganism interactions [6]. Fourth, alterations in microorganism interactions govern the stability of the intestinal ecosystem, which its disruption will contribute to IBD development [7]. Fifth, recent studies have described emerging critical roles of altered trafficking of immune cells and their circuits as crucial drivers of mucosal inflammation in IBD; rather than only resident immune cells and tissue destruction [8]. Despite advances in our understanding of the mechanism of IBD, the specific pathogenic mechanism remains unclear as the clinical treatment of IBD primarily relies on surgery especially in refractory cases and traditional medications that are unable to prevent disease progression and relieve discomfort in IBD patients with decreased quality of life [9]. Based on the above-mentioned circumstances, there is necessity for further exploration of the mechanism of IBD pathogenesis to fulfill unmet medical needs by discovering new approaches to treat this disease (Figure 1).

## 2. Modification-Dependent RNA Function

### 2.1. Methylation System as a Nucleotide Modification

In 1925, around 40 years before the discovery of the DNA double helix structure by Watson and Crick [10], who received the Nobel Prize in Physiology and Medicine in 1962, evidence of the methylation of nucleotides was first reported as pyrimidine 5-methylcytosine (m^5^C) in a study of a nucleic acid constituent in crystalline picrate from hydrolyzed tuberculinic acid [11]. This finding was further confirmed in a study of hydrolyzed thymine [12] and of bacterial and animal viruses with 5-hydroxymethylcytosine (5 hmC), although these studies preliminarily showed the discrimination between DNA and RNA modifications [13,14,15]. In 1958, RNA methylation was clearly confirmed in a study using several sources, including *Escherichia coli*, yeast, rabbit liver, wheat germ, and viruses, which indicate that a methylated thymine base and three methylated adenine bases are generated in RNA [16]. In 1958, the biochemical study showed a natural occurrence of thymine and methylated adenine bases in several RNAs [17].

As a biochemical base of RNA methylation, S-adenosyl methionine (SAM) was identified as an active methyl donor in 1952 [18] and was shown to methylate nicotinamide N-methyltransferase (NNMT), which was partially purified from rat liver in 1951 [19]. Recent studies indicated that the enzymatic activity of NNMT is important for the prevention of nicotinamide (NAM)-mediated inhibition of NAD+-consuming enzymes, such as poly-adenosine-diphosphate (ADP), poly-ADP ribose polymerases (PARPs), and sirtuins (SIRTs) [20]. Increased NNMT activity has been reported in many tumors, including gastrointestinal cancers [21,22,23]. Given that N6-methyladenosine (m6A) is the most prevalent internal RNA modification in messenger RNA (mRNA), a recent study demonstrated that NNMT overexpression resulted in the suppression of m^6^A methylation of CD44 mRNA, a cancer stem-like cell marker, to enhance the formation of CD44v3, a splicing variant. This led to the development of biologically malignant cell phenotypes, such as vascular invasion and distant metastasis in hepatocellular carcinoma, suggesting an association between RNA modification and NNMT [24]. Further, it was reported that kynurenine and NAD+ salvage pathways play a critical role in the successful treatment of IBD, which suggests that potential drug targets include the hydroxycarboxylic acid receptor 3 (HCAR3), a low-affinity receptor for nicotinic acid, and NNMT [25]. Furthermore, it was indicated that enzymes responsible for tryptophan (Trp) degradation via the kynurenine pathway (indoleamine 2,3-dioxygenase 1 [IDO1], kynureninase [KYNU], interleukin-4-induced protein 1 [IL4I1], kynurenine 3-monooxygenase [KMO], and tryptophan 2,3-dioxygenase [TDO2]), receptor of Trp metabolites (HCAR3), and enzymes catalyzing NAD+ turnover (nicotinamide phosphoribosyltransferase [NAMPT], NNMT, PARP9, and CD38) were synchronously coregulated in IBD but not in intestinal malignancies [25]. It was suggested that NNMT helps to maintain a high level of NAD+-dependent proinflammatory signaling by removing excess inhibitory nicotinamide from the biochemical pool.

### 2.2. RNA Modifications

**Figure 2:**
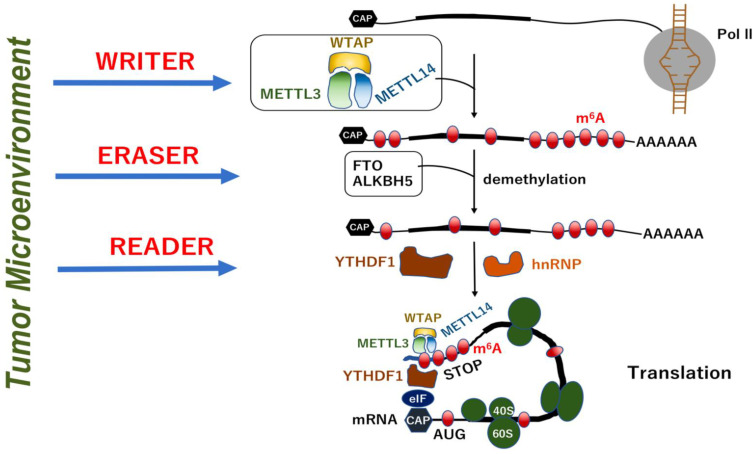
RNAs and their related molecules in human diseases. RNA polymerase-dependent synthesis and modification of mRNAs are modulated in response to the IBD microenvironment. The “writer” proteins (METTL3, METTL14, and WTAP) play a role in mRNA methylation, whereas the “eraser” proteins (FTO and ALKBH5) are involved in the removal of m^6^A methylation marks. These m^6^A marks are interpreted by “reader” proteins, such as Ythdf1, which functions in the peptide translation of mRNAs. This system plays a role in the control of peptide translation in epithelial and immune cells in IBD.

In contrast to DNA modifications, which predominantly occur on m^5^C, m^6^A marks are the most prevalent RNA modifications followed by N1-methyladenosine (m^1^A), m^5^C, and 7-methylguanosine (m^7^G) [26]. The m^1^A modification is analogous to m^6^A, which indicates that their molecular mass is exactly the same and that they cannot be discriminated by mass spectrometry, while m^7^G provides the critical structure of the cap site of mRNA [26]. These epitranscriptomic (RNA modification) are increasingly being appreciated to discriminate among heterogenous cancer types [27]. Further, it was shown that the measurement of m^6^A on microRNAs (miRNAs), which are small non-coding RNAs regulating gene expression, is useful for the detection of early-stage and relapsing cancers [28]. These studies highlight the usage of epitranscriptomic marks as potential diagnostic biomarkers.

m^6^A, which is the most prevalent epitranscriptomic marks on human transcriptome, is catalyzed by N6-adenosine-methyltransferase complex catalytic subunit (METTL3) and methyltransferase-like 14 (METTL14) by forming a stable heterodimer core complex [29]. During the process of RNA methylation, WT1-associated protein (WTAP) interacts with the METTL3-METTL14 complex to affect the methylation reaction [29]. The core complex consisting of METTL3-METTL14 and WTAP plays a role as a “writer” of m^6^A marks [30]. In contrast, several demethylases that reverse this methylation reaction, known as “erasers”, have been identified, which are fat mass and obesity-associated protein (FTO) [31] and α-ketoglutarate-dependent dioxygenase (ALKB) homolog 5 (ALKBH5) [32]. The resultant RNA modifications give rise to binding targets for other proteins, which function as “readers”. The reader proteins include heterogeneous nuclear ribonucleoproteins (hnRNP) and YT521-B homology (YTH) N6-methyladenosine RNA-binding protein 1 (YTHDF1). Interestingly, this RNA modification system is affected by conditions in the extracellular environment, such as hypoxia and inflammation [27], and at least partially by oncogenic signals, as shown by a study on colorectal cancer that the expression of m^6^A reader YTHDF1 is controlled by the oncogene c-MYC [33]. Taken together, previous studies have demonstrated that RNA modification plays a critical role in the homeostasis of colorectal organs, and that, the disruption of this system will result in inflammatory disorders and cancers (Figure 2).

## 3. Modification-Dependent Alterations in RNA Structure

### 3.1. Modification-Dependent RNA Structures

Given that next generation sequencers cannot detect chemical modifications without base substitutions of RNAs, alternative technologies, such as the mass spectrometry (MS) and tunneling-current-based sequencing, are considered as important methods for the analysis of modified nucleosides, especially for miRNAs and tRNAs that play crucial roles in mRNA translation and decoding [26]. Recently, Pytheas, an open-source software package for the automated analysis of tandem MS data for RNA, was described. Pytheas is a very useful analytical tool for sequence characterization of modified RNAs and is expected to facilitate the study of the function and structure of RNA modifications [34]. Recent studies of bacterial tRNA revealed the structural and functional characteristics of the m^6^A modification of RNA and provided two X-ray crystal structures of *Mycoplasma capricolum* TrmM with and without S-adenosyl-l-homocysteine [35]. Although tunneling-current-based sequencing, in which the single-molecule sequential readings are obtained by passing through the nanogap-electrode to determine the nucleotide sequence, can provide nucleotide structures in the absence of enzymatic reactions [27], several recent studies have utilized nanopore-based sequencing methods to detect RNA modifications in their native state. These methods were further developed to be an internal comparison strategy termed “IndoC”, where features, such as “electric current signal intensity” and “raw signal trace meta-information” of potentially modified sites, are identified according to comparisons with other sequences within the sample [[36]. Moreover, another recent study indicated that the technology termed the detection of ribonucleic acid modifications manifested in error rates (DRUMMER) provides a rapid detection of RNA modifications through comparative nanopore sequencing [37]. Taken together, these recently developed analytical methods will be easily transferrable for general use in life science (Table 1).

### 3.2. G-Quadruplexes as Therapeutic Targets in Gastrointestinal Oncogenes

Among DNA repeats in telomeres within chromosomes, the guanine-rich strand (5′-TTAGGG-3′) is lined with four guanines. This strand forms a planar structure called, G-quartet, in the presence of monovalent ions, such as K+, in addition to the double helix [44]. The G-quartet is further layered by an interaction called π-π stacking, which forms a special higher-order structure called the guanine G-quadruplex (G4) [44]. Found in both DNA and RNA, G4 structures are four-stranded nucleic acid molecules that arise from the stacking of G-quartets. In the case of RNA, G-quartets are cyclic arrangements of four guanines of RNA, which are constituted by stacked guanine tetrads held together by Hoogsteen hydrogen bonds that form at key regulatory sites of nucleotides [45]. Although their selective molecular activity has not been fully established, recent studies have indicated that several specific G4 binders can function in the selective inhibition of oncogene expression or specific impairment of telomere maintenance, suggesting the possibility of using them as innovative anticancer drugs [46].

Recent studies have indicated the activity of DNA G4 structures as small molecules against breast cancer by targeting the promoter of the human oncogene c-MYC and telomeres [47]. These small molecules are based on a non-selective thiazole orange scaffold to provide multifaceted interactions with flanking residues [47]. For example, the transcription of the KRAS oncogene is controlled by a G-rich motif, which is located immediately upstream of the transcriptional start site. A recent study shows that hnRNPA1/UP1 can contribute to the unfolding of KRAS G4s to form a regulatory axis for controlling gene expression, suggesting it as a potential new therapeutic target in the rational design of anticancer strategies [48]. Given that oncogenes play roles in gastrointestinal cancer, further research in this direction might yield a discovery of innovative therapeutic targets.

## 4. RNA Modifications in IBD

### 4.1. Modification of tRNAs in IBD

**Figure 3:**
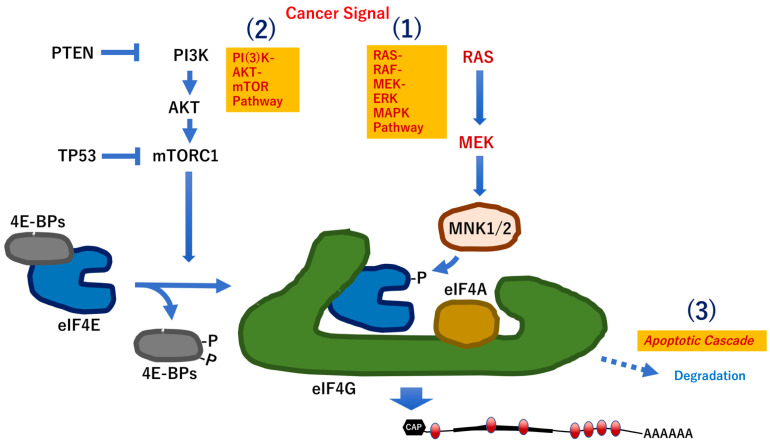
RNA modification mechanism in IBD. Generally, three mechanisms are involved in the activation of peptide translation of mRNAs. PTEN, phosphatase and tensin homolog; TP53, Tumor Protein P53; AKT, AKT serine/threonine kinase 1; mTORC1, mechanistic/mammalian target of rapamycin complex 1; eIF4E, eukaryotic translation initiation factor 4E; 4EBP, eukaryotic translation initiation factor 4E binding protein; eIF4G, eukaryotic translation initiation factor 4 gamma; eIF4A, eukaryotic translation initiation factor 4A; MNK1, MAPK interacting serine/threonine kinase 1; MNK2, MAPK interacting serine/threonine kinase 2; MAPK, mitogen-activated protein kinase; ERK1, mitogen activated protein kinase 3 (MAPK3); ERK2, mitogen activated protein kinase 1 (MAPK1); MEK1, MAP2K1, mitogen-activated protein kinase kinase 1; RAS, RAS proto-oncogene, GTPase; RAF, RAF-1 proto-oncogene, serine/threonine kinase.

Recent studies have indicated that alterations in chemical modifications (e.g., methylation) in transfer RNA (tRNA)-derived fragments, such as tRNA-derived RNA fragments (tRFs) and tRNA-derived stress-induced RNAs (tiRNAs), are emerging as new research hotspots [49]. These alterations in tRNAs play essential roles not only in peptide translation but also in cellular homeostasis, such as cell migration, proliferation, and apoptosis, which are critical to the pathology of mucosal immunity and its related disease, IBD [49] (Figure 3). Given that the RNA polymerase III enzyme produces tRNA, a ubiquitous nucleic acid that synthesizes precursor tRNAs that undergo maturation processes before exportation to the cytoplasm, tRNAs have additional biological functions and act as non-ribosomal substrates [50]. In this case, tRFs are derived from mature or primary tRNAs [51]. The tRFs are classified into five types according to their derivations: tRF-1, tRF-2, tRF-3, tRF-5, and i-tRF [51]. Cleavages in the D-loop of tRNAs give rise to 5′-tRFs, whereas those in the T-loop of tRNAs produce 3′-tRFs [52]. On the contrary, certain stress conditions (e.g., oxidative stress, hypoxia, heat shock, and infection) induce the production of tiRNAs, 5′-tiRNAs, and 3′-tiRNAs from the anticodon loop regions in tRNAs [53]. During biogenesis of these tRNA derivatives, the following two enzymes are involved: (1) Rny1p, a member of the T2 family of ribonucleases [54]; and (2) angiogenin, a member of the ribonuclease A family [55]. The biological functions of tRFs and tiRNAs are similar, which they can both inhibit translation initiation and elongation of peptides; thus, they are regarded as actionable cellular factors [50].

As a member of the eukaryotic translation initiation factor 2A (eIF2α) family of protein kinases that inhibit translational initiation in response to stress stimuli, protein kinase RNA-dependent (PKR) functions as a key mediator of the interferon-induced antiviral response [56]. After PKR binds to double-stranded (ds)RNA, the PKR protein can change its conformation to form a dimer complex. This process activates PKR through phosphorylation and dimerization [56,57]. Studies have indicated that modifications of tRNA nucleosides (e.g., m^5^C, 2-thiouridine, and m^1^A) suppress PKR activation in cells, whereas unmodified or lightly modified (fewer than three nucleosides per one tRNA copy) tRNA can activate PKR [56,57]. These reports suggest that the formation of tRFs and tiRNAs can define cellular function through PRK kinase activity.

Previous reports demonstrated that aberrant processing of human tRNAs can induce a mucosal immune response. For example, sideroblastic anemia with B-cell immunodeficiency, fever, and developmental delay (SIFD) is an inherited recessive disease with a mutation in tRNA nucleotidyltransferase 1 (TRNT1) [58]. It has been proposed that a TRNT1 abnormality results in decreased expression of mature tRNAs, which can disrupt peptide synthesis homeostasis, as they act as triggers to stimulate the innate immune response in various tissues including mucosal epithelium [59].

**Figure 4:**
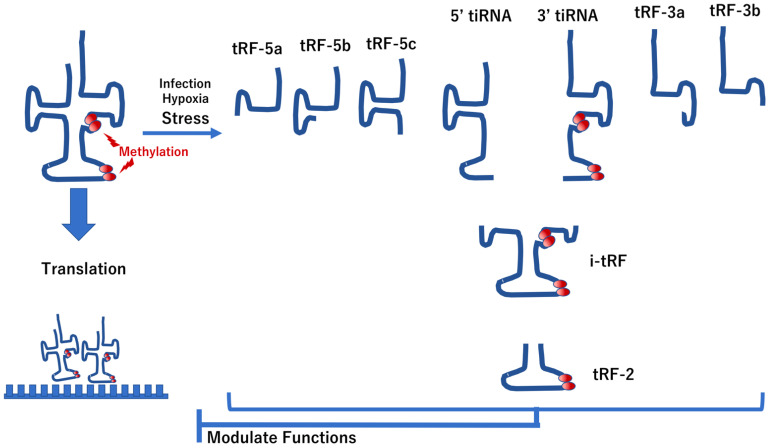
RNAs, their modification and their functions in stress. Under stress (e.g., hypoxia, viral or bacterial infections), the production of tRNA-derived stress-induced RNAs (e.g., 5′-tiRNA and 3′-tiRNA) occurs via enzymatic cleavage of tRNAs. This cleavage also leads to the generation of tRNA-derived RNA fragments (tRFs), which are involved in the modulation of translation function of standard tRNAs. Several positions in tRNAs are methylated, where such methylation modulates tRNA functions.

Recent studies have indicated that tRFs regulate cell proliferation as tRFs are fragments of tRNAs and share some similarities with other nucleotides, such as miRNAs. The biological functions of tRFs include translational regulation and gene silencing, which are critical processes in the occurrence and development of inflammation and cancers [60]. Recently, another study verified that tRF/*miR-1280*, a fragment of tRNALeu and pre-miRNA, suppresses stem cell-like cells and metastasis by repressing the jagged canonical notch ligand 2 (JAG2)-notch signaling pathway [61], suggesting that a network of tRF and miRNAs regulate cellular function. Previous studies showed that tRF/*miR-1280*-related tumors were associated with mucosal immunity via innate lymphoid cells, including natural killer (NK) cells and innate lymphoid cells (ILC1, ILC2, ILC3), which are innate immune system cells derived from common lymphoid progenitor cells. These cells secrete signaling molecules in response to pathogenic tissue damage and control both innate and adaptive immune responses [62]. It was also shown that tiRNAs may be involved in apoptosis through interaction with cytochrome c, which inhibits the induction of apoptosis [63]. It is possible that targeting the mechanism of tiRNA may modulate IBD phenotypes as tiRNAs are involved in the regulation of apoptosis induction. Taken together, tRNAs can be considered as possible druggable targets for novel treatment approaches for IBD (Figure 4).

### 4.2. Modification of mRNA in IBD

Recent studies have revealed the implication of m^6^A mRNA methylation in susceptibility to IBD. Interestingly, given that single nucleotide polymorphisms (SNPs) located near or within m^6^A motifs have been proposed as possible contributors to disease, a recent study identified five candidate genes corresponding to two of the major IBD subtypes: ubiquitin-conjugating enzyme E2L3 (*UBE2L3*) and solute carrier family 22 member 4 (*SLC22A4*) for Crohn’s disease and transcription factor 19 (*TCF19*), chromosome 6 open reading frame 47 (*C6orf47*), and small nuclear RNA-activating complex polypeptide 4 (*SNAPC4*) for ulcerative colitis [64]. UBE2L3 functions in the ubiquitination of tumor protein P53 (TP53), the proto-oncogene c-FOS, and the NF-kappa-B (NF-kB) precursor p105 [65]. SLC22A4 functions as a polyspecific organic cation transporter, which is critical for elimination of many endogenous small organic cations and a wide array of drugs and environmental toxins [66], while SNAPC4 is associated with spondylitis [67]. Another study indicated that a broad landscape of m^6^A modifications is altered in IBD, while m^6^A regulators displayed extensive differential expression, including that of some shared common differentially expressed genes [68]. However, a single-cell-based analysis is necessary for a complete understanding of cell-to-cell communications and the discovery of novel therapeutic targets using m^6^A marks.

### 4.3. Modification of ncRNAs in IBD

Given that m^6^A, m^1^A, m^5^C, and m7G are the major forms of RNA methylation modifications, a recent study presented evidence of epitranscriptomic marks on long non-coding RNAs (lncRNAs, any non-coding RNAs longer than 200 nucleotides) in colorectal cancers and described a methylation-related lncRNA signature for predicting “hot tumors” with a better prognosis and “cold tumors” with a poorer prognosis [69]. Another study of colorectal cancer indicated that the expressions of two lncRNAs, *AC156455.1* and *ZEB1-AS1*, were increased in the high-risk group of cervical cancer. A signature of seven m^6^A-related lncRNAs can independently predict disease prognosis and is closely associated with immune cell infiltration, suggesting new therapeutic targets for disease control [70]. Although evidence related to lncRNAs and IBD is scarce, presumably due to the limitation of methods that can measure precise sites of RNA modifications in lncRNAs, more experiments are required to identify which lncRNAs may be novel therapeutic opportunities for mucosa-related cancer in IBD.

### 4.4. Modifications of miRNA in IBD

Reported as variations with respect to the reference sequence of miRNAs in the miRbase database [71], isomiRs are sequence variations with respect to the reference sequence and newly discovered molecules in cancer and other diseases. IsomiRs contain variations in their 3′- or 5′-termini, and, less frequently, nucleotide substitutions along the miRNA length, which are responsible for the active role of isomiRs [72]. Although little is known about the role of isomiRs and the interactions of miRNA with non-canonical targets in IBD, recent reports have described that the biogenesis and function of miRNAs are altered under various stresses, including chronic stress, inflammation, and age-associated neurodegenerative disorders [73]. More studies are needed to elucidate the possible involvement of isomiRs in chronic disorders such as IBD.

## 5. RNA Modifications That Regulate IBD Phenotypes

Although extensive efforts have been made to investigate epigenetic modifications in IBD to clarify how epigenetic mechanisms regulate IBD [74], little evidence of RNA modifications in IBD has accumulated. IBD-related cancers differ from those that harbor numerous mutations, i.e., deletions, insertions, or substitutions of nucleotide(s). More importantly, previous reports have noted that genetic factors provide limited indications of the mechanism of IBD, which is similar to other autoimmune diseases, such as psoriasis and rheumatoid arthritis [75]. It has been well demonstrated that non-genetic factors, i.e., epigenetics, play roles in the regulation of in IBD, given that they can contribute to gene expression without changing the genetic code, although the underlying specific mechanism remains unclear.

### 5.1. RNA Modification-Dependent Alterations in IBD Phenotypes

mRNA modification of adenosine (e.g., m^6^A) is an important modulator of RNA stability and decay as well as protein translation on ribosomes. Recent studies have indicated that the m^6^A modification is essential for maintaining homeostatic self-renewal of colonic stem cells by the regulatory function of the methyltransferase 14 (Mettl14) gene in mice [76] and in whole tissues by the methylation-reading functions of the following common differentially expressed genes in humans: insulin-like growth factor 2 mRNA-binding protein 2 (IGF2BP2), heterogeneous nuclear ribonucleoprotein A2/B1 (HNRNPA2B1), zinc finger CCHC-type containing 4 (ZCCHC4), and eukaryotic translation initiation factor 3 subunit I (EIF3I) [68]. This evidence indicates significant participations of m^6^A enzymes in multiple physiological and pathological processes. It is suggested that the m^6^A modification may be a potential therapeutic target in IBD, considering that oncogenic signals, such as PI(3)K-AKT-mTOR pathway and RAS-RAF-MEK-ERK MAPK pathway, can be control by a m^6^A modification-dependent translation, and the m^6^A mRNA modification maintains epithelial cell homeostasis via NF-κB-mediated antiapoptotic pathway in bowel [76]. Moreover, previous studies have demonstrated the emerging crucial role of RNA modifications in intestinal mucosal immunity via the regulation of RNA function, and thus, these modifications are closely related to the occurrence of IBD and the subsequent development of colorectal cancer. Therefore, m^6^A-related genes and regulatory factors are expected to be potential predictive markers and therapeutic targets against inflammation and cancer [77].

### 5.2. RNA Modification Control of T-Cell Homeostasis

The involvement of RNA modifications in T-cell homeostasis was first reported in a study of the IL-7/STAT5/SOCS pathways [78]. This study demonstrated that the mRNAs of the suppressor of cytokine signaling (SOCS) gene family were marked by m^6^A enzymes, which led to slower mRNA decay and increased mRNA in Mettl3-deficient naive T cells. Further, the increased SOCS gene family activity consequently inhibited IL-7-mediated signal transducer and activator of transcription 5 (STAT5) activation and T-cell homeostatic proliferation and differentiation [78]. It has also been reported that the proinflammatory background due to SOCS1 deficiency induces dysbiosis of the gut microbiota, which in turn results in a procolitogenic environment in a mouse model of IBD [79]. In addition, it was shown that SOCS2 deletion protects against damage in dextran sodium sulfate-induced IBD in mice [80]. The optimal activation of STAT5 is required for IL-22 production in group 3 innate lymphoid cells (ILC3) in colitis, which is critical for the homeostatic maintenance of gut epithelial integrity during *Citrobacter rodentium*-mediated colitis-induced intestinal disease, suggesting the potential importance of STAT5 in other idiopathic forms of colitis, such as IBD [81]. These studies indicated that appropriate signaling by the SOCS family is necessary for homeostasis in intestinal organs; the disruption of which results in colitis, such as IBD, in mice. However, the significance of RNA modifications in the IBD remain to be understood completely. Studies of these systems in humans are important for early diagnosis and for monitoring the remission and recurrence of IBD and will also be beneficial for the development of innovative medicines to control IBD.

### 5.3. RNA Modifications Control Immune Checkpoints

It was reported that RNA modifications regulate immune responses [82]. One study demonstrated a negative correlation between METTL3 or METTL14 and STAT1 in patients with low mutational tumor burden and tumors associated with the mismatch-repair-proficient or microsatellite instability-low (pMMR-MSI-L) genotypes. The inhibition of RNA modification by depletion of METTL3 and METTL14 enhanced response to anti-programmed death receptor-1 (PD-1) treatment in pMMR-MSI-L colorectal cancer and melanoma [82]. It was proposed that this improved efficacy is based on the mechanism, which the depletion of METTL3 and METTL14 causes the stabilization of STAT1 and interferon regulatory factor 1 (IRF1) mRNA via YTHDF2, leading to interferon (IFN)-γ signaling and increased numbers of cytotoxic tumor-infiltrating CD8+ T cells in vivo. This cascade of events provides the immunologically “hot” condition and sensitivity to exposure to anti-PD-1 treatment [82]. The study of erasers of RNA modifications demonstrated that m^6^A demethylases contribute to the efficacy of immunotherapy and identify ALKBH5 as a potential therapeutic target to enhance immunotherapy outcomes in melanoma and colorectal cancer [83]. It is suggested that RNA modifications may be targets for new potential anticancer immunotherapies, although any involvement of RNA modifications in IBD remain to be investigated. Importantly, given that RNA modification is a potential target in established tumors with mutational burdens and immunologically hot conditions, it remains unclear whether targeting RNA modifications can reverse early phases of carcinogenesis with few genetic mutations. Moreover, it remains unclear whether targeting these modifications could be useful for the chemical prevention of cancer and the treatment of precancerous lesions or colitis, such as IBD. To this end, reagents that target RNA modifications will be subjected to clinical trials for testing in several human diseases [84] (Table 2).

## 6. Conclusions

**Figure 5:**
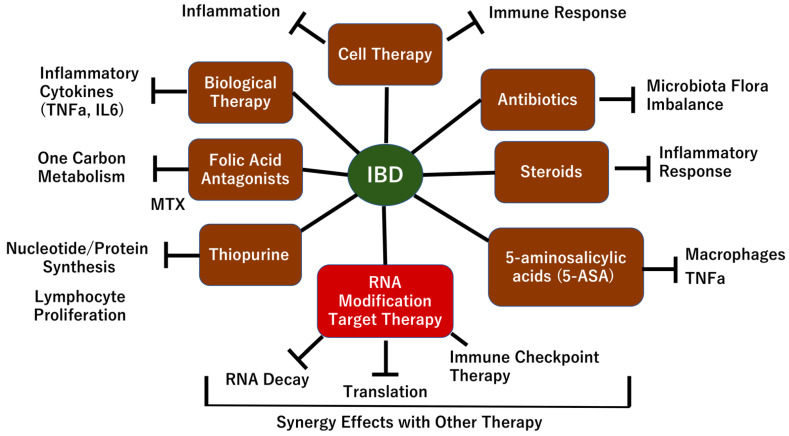
Therapeutic targets of RNA modifications in IBD. In IBD, several therapeutic approaches (e.g., biological therapy, folic acid antagonist, thiopurine, 5-aminosalicylic acids (5-ASA), and steroids) have been developed to treat inflammation to control the excess reaction of the inflammatory mechanism. To control bacterial flora, antibiotics are used to inhibit or reduce pathological organisms. Cell therapy may modulate the inflammatory response of IBD. RNA modifications can be targets for the IBD mechanism to regulate altered translation and RNA processing of splicing and decay in epithelial and immune cells, which will exert synergistic effects with immune checkpoint inhibitor therapy.

The chemical modifications without base substitutions of RNAs cannot be detected by standard next generation sequencers, and thus, a special sequencing technique suitable for this purpose is required. The results of previous studies strongly suggest that RNA modifications play specific roles in the pathophysiology of IBD. To elucidate this mechanism and develop breakthrough diagnostics and innovative therapies, it is necessary to clarify the phenomenon of RNA modifications at the single-cell level. By doing so, it is possible to enhance the effects of standard genomic medicine, molecular-targeted therapy, and cancer immunotherapy that are currently being administered and to substantially contribute to the development of precision medicine (Figure 5).

## Figures and Tables

**Table 1:** Recently developed analysis tools for RNA modifications.

Tool	Description	Merits and Applications	References
IndoC	“Trace” and “current signal intensity” of potentially modified sites are compared	- Data are validated by the interaction of chemical probe adduct. - This allows separation from high mismatch sites that display single nucleotide polymorphism-like behavior.	[36]
DRUMMER	A rapid detection of RNA modifications through comparative nanopore sequencing	- This has similar sensitivity to signal-level analysis approaches and correlates well with orthogonal approaches.	[37]
EpiNano	Detection of m^6^A RNA modifications using Oxford nanopore direct RNA sequencing	- Using Guppy, a data processing toolkit that contains Oxford Nanopore’s base-calling algorithms, this tool can predict the m^6^A sites directly from RNA nanopore sequencing datasets. - This tool takes FASTQ as input data format and outputs the data in FAST5 data format. - This tool can correct base-calling “error” features.	[38]
Quantum Sequencer System	Sequencing and mapping tool of RNA base modifications in microRNAs, such as m^6^A or m^5^C	- Evaluation of the methylation ratio of cytidine and adenosine at each site in single-molecule sequences. - This tool supports the study of cancer cell propagation and suppression.	[39]
HPC-REDItools	A novel HPC-aware tool for improved large-scale RNA-editing analysis	- This tool is upgraded for accurate RNA-editing events discovered in large dataset repositories.	[40]
RED-ML	An effective RNA-editing detection method based on machine learning	- This is a highly accurate, rapid, and general-purpose tool for RNA-editing detection using RNA sequencing data.	[41]
RNAEditor	An easy method for the detection of RNA-editing events and the introduction of editing islands	- This tool provides the Graphical User Interface (GUI), allowing for an easy-to-use analysis of RNA-seq data.	[42]
CoverageAnalyzer (CAn)	A tool for inspection of modification signatures in RNA sequencing profiles	- This is an intuitive tool that includes the simple visualization of RNA sequencing data and RNA modification analysis.	[43]

**Table 2:** Reagents targeting RNA modifications.

Regents	Target(s) and Action	Diseases	Expected Trials	References
STM2457	Inhibitor of METTL3 (and METTL14)	Myeloid leukemia	Phase I	[85]
UZH1	METTL3	Leukemia	SC	[86]
CS1, CS2	FTO inhibitor	Leukemia and solid tumors	SC	[87]
FB23	FTO inhibitor	Leukemia	SC	[88]
R-2-hydroxyglutarate (R-2HG)	FTO inhibitor	Glioma and leukemia	SC	[89]
S-adenosyl-homocysteine	Inhibitor of METTL3 and METTL14	Metabolic diseases	Phase I	[90]
MO-I-500	FTO inhibitor	Breast cancer	SC	[91]
Meclofenamic acid	FTO inhibitor	Glioblastoma	Phase I	[92]
I-BET	Bromodomain and extra-terminal (BET) protein inhibitors	Leukemia	Phase I	[93]

SC, subclinical.

## Data Availability

Not applicable.
